# The performance of digital technologies for measuring tuberculosis medication adherence: a systematic review

**DOI:** 10.1136/bmjgh-2024-015633

**Published:** 2024-07-16

**Authors:** Miranda Zary, Mona Salaheldin Mohamed, Cedric Kafie, Chimweta Ian Chilala, Shruti Bahukudumbi, Nicola Foster, Genevieve Gore, Katherine L Fielding, Ramnath Subbaraman, Kevin Schwartzman

**Affiliations:** 1 McGill International Tuberculosis Centre, Research Institute of the McGill University Health Centre, Montreal, Quebec, Canada; 2 TB Centre, London School of Hygiene and Tropical Medicine, London, UK; 3 Department of Public Health and Community Medicine, Tufts University School of Medicine, Boston, Massachusetts, USA; 4 McGill Schulich Library of Physical Sciences, Life Sciences and Engineering, McGill University, Montreal, Quebec, Canada; 5 Division of Geographic Medicine and Infectious Diseases, Tufts Medical Center, Boston, Massachusetts, USA

**Keywords:** Systematic review, Tuberculosis, Global Health, Public Health

## Abstract

**Introduction:**

Digital adherence technologies (DATs), such as phone-based technologies and digital pillboxes, can provide more person-centric approaches to support tuberculosis (TB) treatment. However, there are varying estimates of their performance for measuring medication adherence.

**Methods:**

We conducted a systematic review (PROSPERO—CRD42022313526), which identified relevant published literature and preprints from January 2000 to April 2023 in five databases. Studies reporting quantitative data on the performance of DATs for measuring TB medication adherence against a reference standard, with at least 20 participants, were included. Study characteristics and performance outcomes (eg, sensitivity, specificity and predictive values) were extracted. Sensitivity was the proportion correctly classified as adherent by the DAT, among persons deemed adherent by a reference standard. Specificity was the proportion correctly classified as non-adherent by the DAT, among those deemed non-adherent by a reference standard.

**Results:**

Of 5692 studies identified by our systematic search, 13 met inclusion criteria. These studies investigated medication sleeves with phone calls (branded as ‘99DOTS’; N=4), digital pillboxes N=5), ingestible sensors (N=2), artificial intelligence-based video-observed therapy (N=1) and multifunctional mobile applications (N=1). All but one involved persons with TB disease. For medication sleeves with phone calls, compared with urine testing, reported sensitivity and specificity were 70%–94% and 0%–61%, respectively. For digital pillboxes, compared with pill counts, reported sensitivity and specificity were 25%–99% and 69%–100%, respectively. For ingestible sensors, the sensitivity of dose detection was ≥95% compared with direct observation. Participant selection was the most frequent potential source of bias.

**Conclusion:**

The limited number of studies available suggests suboptimal and variable performance of DATs for dose monitoring, with significant evidence gaps, notably in real-world programmatic settings. Future research should aim to improve understanding of the relationships of specific technologies, settings and user engagement with DAT performance and should measure and report performance in a more standardised manner.

**WHAT IS ALREADY KNOWN ON THIS TOPIC**

- Several cohort studies have suggested that digital adherence technologies (DATs) can both underestimate and overestimate medication ingestion among persons treated for tuberculosis. No previous review has synthesised available evidence in this regard.

**WHAT THIS STUDY ADDS**

- Reports of DAT (medication sleeves with phone calls, digital pillboxes) implementation in real-world treatment settings consistently indicate suboptimal performance for measuring medication adherence. However, available evidence is limited in scope and quality.

**HOW THIS STUDY MIGHT AFFECT RESEARCH, PRACTICE OR POLICY**

- Suboptimal dose reporting from DATs potentially compromises their effectiveness and programme efficiency. Future clinical practice will be strengthened by rigorous technology evaluations that reflect more consistent use of reference standards and clearer benchmarks for medication adherence.

## Introduction

 Tuberculosis (TB) disease requires treatment with medication regimens involving varying pill burdens lasting at least 4 months. Adherence to these treatment regimens—which involve daily medication intake—is crucial, as non-adherence can lead to treatment failure, relapse, development of drug resistance, ongoing TB transmission and death.^1 2^ Treatment for TB infection lasts between 1 and 9 months. Adherence to treatment for TB infection is essential to reduce the risk of developing TB disease but is often difficult to achieve in routine care, particularly since treated persons are asymptomatic.^3 4^ Directly observed therapy (DOT) has been recommended for TB treatment support.^5^ DOT typically involves healthcare workers or community workers, watching up to 100% of prescribed medication doses.^6^ However, DOT is logistically challenging, expensive, potentially intrusive, raises ethical concerns and can have limited or varying effectiveness for improving treatment outcomes.^6^,^8^

Digital adherence technologies (DATs) have been increasingly studied and used in routine care as an alternative or adjunctive approach for supporting TB treatment. DATs include mobile communication and other innovations that can remind people with TB to take their medication, digitally observe doses taken, compile dosing histories, triage people who may be at higher risk for unfavourable treatment outcomes and enable differentiated (ie, intensified or individualised) care.^2^ DATs include a range of technologies that may facilitate more person-centred approaches for monitoring adherence, potentially improving treatment outcomes.^2^

For example, one of the most widely used DATs, branded as 99DOTS, involves wrapping a paper sleeve over a medication blister pack. Dispensation of a medication dose then reveals a hidden phone number. By calling this number, the person with TB can report dose ingestion, creating a digital dosing history that allows early identification by healthcare providers of people who may be non-adherent.^9^,^12^ However, people with TB may call the phone number on their medication sleeve or open an electronic pillbox without ingesting medication (ie, over-reporting adherence). They can also ingest medication doses without calling the designated phone number, or after removing multiple doses from their electronic pillbox (ie, under-reporting adherence). Over-reporting can reflect a desire to report optimal adherence, that is, social desirability bias.^13^ Under-reporting can be influenced by inadequate accessibility of or training on the technology, resulting in limited engagement.^14^,^17^ With the potential exceptions of video-observed treatment and ingestible sensors, none of the other DATs measures ingestion directly. Hence for most DATs, some degree of measurement error is expected.

An initial systematic review examining DATs for TB treatment support was published in 2018.^18^ However, since 2018—and particularly with the COVID-19 pandemic—interest in and experience with these technologies have expanded substantially.^18^ Other reviews have been published subsequently. However, they often focused on specific technologies,^19^ study types^20^ or outcomes (ie, acceptability).^21^ None has examined the performance of DATs for measuring TB medication adherence, which is crucial for healthcare providers. If DATs do not yield accurate assessments of adherence, any resulting data are of questionable value, resulting in limited public health impact.^2^ The present systematic review synthesises evidence on the performance of DATs for measuring TB medication adherence, among persons treated for TB disease and infection.

## Methods

### Design

Our systematic review protocol was registered in PROSPERO, the International Prospective Register of Systematic Reviews (CRD42022313526).^22^ This review follows the Preferred Reporting Items for Systematic Reviews and Meta-Analyses (PRISMA) guidelines. The PRISMA checklist is available in online supplemental materials.

### Search and screening strategy

The search for relevant literature was conducted on 28 April 2023 (updated from 14 April 2022) in MEDLINE/Ovid, Embase, CENTRAL, CINAHL, and Web of Science Core Collection, plus Europe PMC preprints (including MedRxiv) and ClinicalTrials.gov, from 1 January 2000 to 28 April 2023. Key search concepts included TB (disease or infection), digital technologies (such as mobile phone, smartphone, video observation, digital pillboxes and text messaging) and accuracy (such as sensitivity, specificity and area under the curve (AUC)). The complete search strategy can be found in online supplemental table S1. The database searches were conducted by a health librarian (GG). Separately, we handsearched the Union World Conference on Lung Health for relevant abstracts on DATs and performance from 2004 to 2022 inclusively, as this is the major venue for public health-oriented TB research. There were no language restrictions.

### Inclusion/exclusion criteria

Studies were included if they reported a quantitative outcome addressing the performance of DATs for measuring TB medication adherence (ie, any of sensitivity, specificity, positive predictive value (PPV), negative predictive value (NPV), AUC, likelihood ratio, accuracy or agreement). These are defined below (table 1). Articles were included if the number of participants using the DAT was at least 20, and the study design included the comparison of adherence reports generated by a DAT with a reference standard such as urine drug metabolite testing, pill count, direct observation of medication ingestion or other such information. DAT interventions included but were not limited to smartphone-based technologies such as phone-based dosing records, short message service (SMS) or video-supported treatment, digital pillboxes and ingestible sensors. We defined a DAT as an intervention with a digital component (which could be part of a multicomponent intervention) with the intention to measure and promote treatment adherence and/or reduce missed visits and/or reduce losses to follow-up. Examples of DATs included and excluded with this definition can be found in online supplemental table S2.

**Table 1:** Definitions of parameters used to describe the performance of digital adherence technologies (DATs) for measuring tuberculosis medication adherence

Performance Parameter	Definition—dose	Definition—person
Sensitivity	The percentage of doses that were classified as taken by the DAT, among those that were taken according to the reference standard.	The percentage of participants who were classified as adherent by the DAT, among those deemed adherent by the reference standard.
Specificity	The percentage of doses that were classified as not taken by the DAT, among those that were not taken according to the reference standard.	The percentage of participants who were classified as non-adherent by the DAT, among those deemed non-adherent by the reference standard.
Positive predictive value	Likelihood of a dose being taken according to the reference standard among those deemed as taken by the DAT.	Likelihood of a participant being deemed adherent by the reference standard among those classified as adherent by the DAT.
Negative predictive value	Likelihood of a dose not being taken according to the reference standard among those classified as not being taken by the DAT.	Likelihood of a participant being deemed non-adherent by the reference standard among those classified as non-adherent by the DAT.
Accuracy	The percentage of doses that were classified as taken or not taken by both the DAT and the reference standard.	The percentage of participants who were classified as adherent or non-adherent by both the DAT and the reference standard.

Parameters are defined against a reference standard and by a unit of assessment.

We included studies of persons treated for TB disease or infection, including subgroups such as people with drug-resistant TB and people with HIV. Eligible study designs included all observational studies except case–control studies.^23^ We excluded reports if they did not, in fact, involve a DAT (online supplemental table S2), or if they were reviews, editorials, commentaries, news articles or study protocols. Abstracts were excluded other than those presented at the Union World Conferences on Lung Health. Relevant grey literature (such as ministry reports, technical papers and preprints) was accepted if it met the eligibility criteria. A complete list of the outcomes, population, intervention and control groups of interest, and all inclusion/exclusion criteria can be found in online supplemental table S3.

### Study selection

After deduplication using EndNote (V.20.2.1—Clarivate, London UK), five reviewers (MZ, MSM, CIC, SB, CK and NF) independently screened all titles and abstracts for their relevance to DATs for TB treatment support, supported by Rayyan.ai (Rayyan, Cambridge, USA).^24 25^ Potentially relevant studies underwent independent full-text review by the same reviewers, for eligibility according to the inclusion criteria above. Each screening stage was conducted in duplicate, by two reviewers blinded to each other’s assessment, with conflicts resolved by a senior investigator (KS, RS or KLF). All references from and citations of each included publication were also screened for inclusion using Google Scholar (Alphabet, Mountain View, USA).^26^

### Data extraction

For each included study, data were extracted into a prespecified Excel (Microsoft, Redmond, USA) template by two independent reviewers (MZ and MSM or CK) in parallel, and subsequently compared for any discrepancies. Conflicts were resolved by consensus and discussion with a third reviewer (KS) when necessary. Extracted data included study characteristics, for example, study design, study setting (ie, geographic location; inpatient or outpatient), participant characteristics, DAT used, reference standard used, the approaches to classifying adherence for both the DAT and reference standard (eg, time frame and frequency of adherence assessment), and any important gaps noted by the reviewers. For each study, we extracted all reported performance parameters for example, true positives, true negatives, false positives, false negatives, sensitivity, specificity, PPV, NPV, accuracy and AUC.

### Performance parameter definitions

The performance of digital technologies for measuring TB medication adherence can include an assessment of the performance of the DAT for detecting human behaviour or the technical performance or functioning of the DAT. The performance for detecting human behaviour is assessed in real-world conditions, to determine its ability to detect a person’s adherence to their medication during their treatment course. Technical performance is assessed in controlled conditions to determine whether the designated hardware or software of the DAT can adequately detect specific doses known to have been taken. In either condition, DAT performance can be assessed per person or per medication dose. Table 1 defines the performance parameters used in this review by the unit of assessment: dose versus person.

### Data synthesis

The extracted data were summarised in tabular form. Prespecified subgroup analyses were performed where appropriate, addressing specific DATs, TB disease versus infection and groups at risk of unfavourable outcomes. Sensitivity and specificity estimates were displayed according to DAT type in forest plots created using RevMan (V.5.4—Cochrane, London UK).^27^ We calculated pertinent performance parameters that were not directly reported when these could be derived from the underlying data. For parameters that were reported without binomial 95% CIs, they were calculated, when possible, using the Clopper-Pearson Exact Method in R (V.4.1.2—GNU Project).^28^ This method was also used for studies that involved repeated observations per individual, and therefore, does not account for within-individual clustering.

Publication bias was assessed qualitatively using Deek’s test for diagnostic accuracy studies.^29^ We created a funnel plot of the association between the diagnostic OR (DOR) and the effective sample size (ESS) of each study.^29^ Quantitative assessment for publication bias using the associated regression test of asymmetry could not be performed, given the small number of included studies.

### Quality assessment

The included articles were assessed for risk of bias and applicability concerns using the Quality Assessment of Diagnostic Accuracy Studies 2 (QUADAS-2) tool for primary diagnostic accuracy studies.^30^ This tool addresses participant selection, the performance of the index test, the performance of the reference test, and the flow and timing of the tests. For each feature, three scores could be used: low, unclear or high. Two reviewers (MZ and MSM or CK) independently assessed each study for quality. Any conflicts were resolved by consensus and discussion with a third reviewer (KS) when necessary. Quality assessment results are displayed in graphical and summary format using RevMan (V.5.4—Cochrane, London UK).^27^

### Grading of Recommendations, Assessment, Development and Evaluation assessment

Finally, we rated the robustness of evidence using the Grading of Recommendations, Assessment, Development and Evaluation (GRADE) approach for diagnostic tests and strategies.^31^ The GRADEpro Guideline Development Tool (McMaster University, Canada & Evidence Price, EU) was used for recommendations.^32^ The risk of bias and indirectness was assessed using the relevant elements of the QUADAS-2 checklist. Inconsistency and imprecision were assessed based on outcome variability and CI ranges across articles.

### Patient and public involvement

Patients and the public were not specifically involved in the design, conduct, reporting or dissemination plans of our research.

## Results

### Study selection

**Figure 1:**
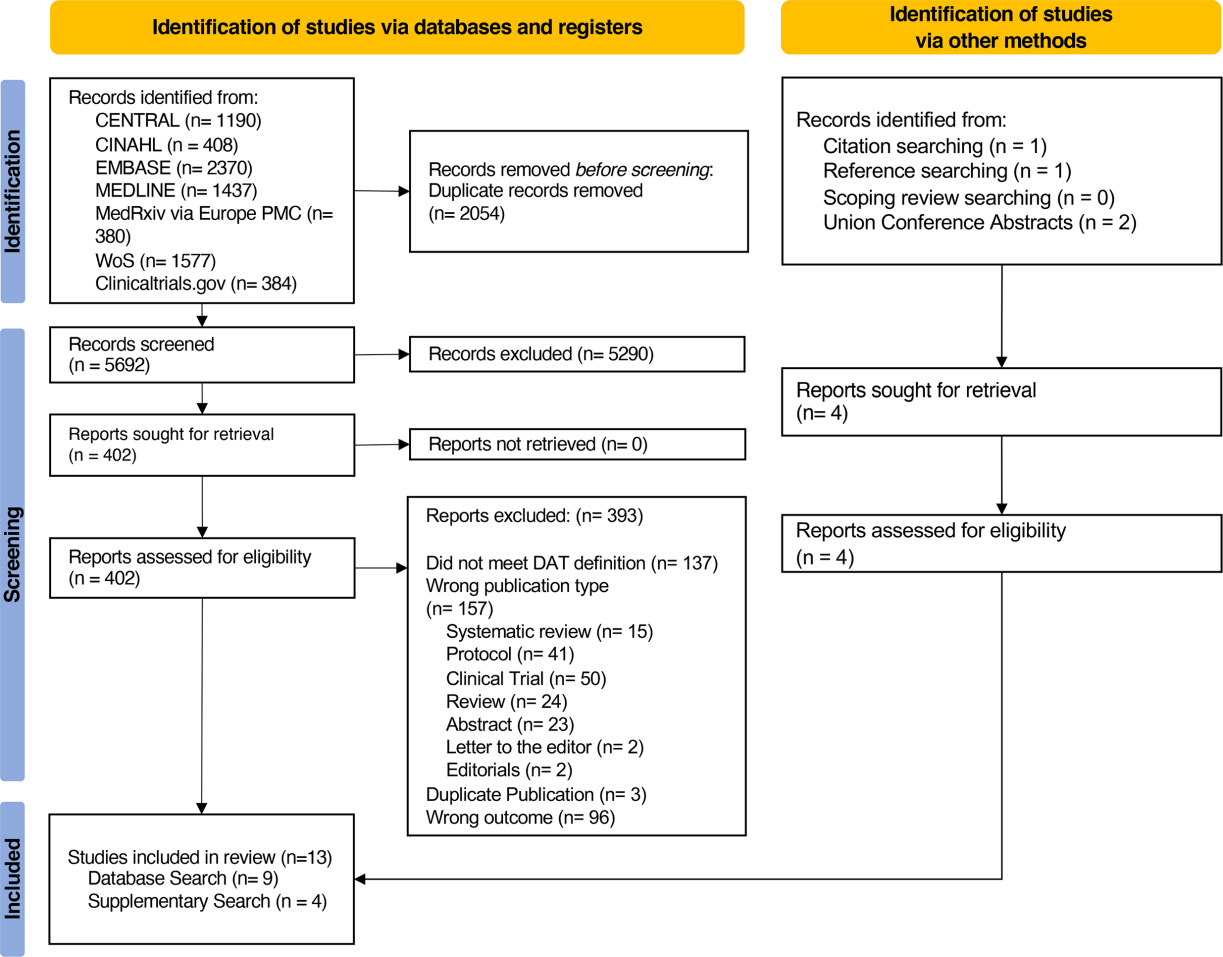
PRISMA 2020 flow diagram of studies identified and included. Studies were identified via databases and registries, and via other methods related to the performance of digital adherence technologies (DATs) for measuring tuberculosis (TB) medication adherence. Other methods included searching the references and citations of included studies identified from databases and registries, searching the references and citations of the studies included in our linked systematic reviews and searching the abstracts of the Union World Conferences on Lung Health from 2004 to 2022. Database and registry searches were conducted on 28 April 2023 from 1 January 2000 to 28 April 2023. PRISMA, Preferred Reporting Items for Systematic Reviews and Meta-Analyses.

Figure 1 illustrates the PRISMA 2020 flow chart. After deduplication, there were 5692 records identified, of which 5290 titles and abstracts were not relevant to TB and DATs. Of the remaining 402 reports which underwent full-text review, 9 met our inclusion criteria. Four additional reports were identified by handsearching, for a total of 13 included reports.

### Overview of studies

Of the 13 reports included, 4 involved medication sleeves with phone calls, 5 involved digital pillboxes, 2 involved ingestible sensors and 1 each involved a mobile application and an artificial intelligence (AI)-based technology for viewing videos of medication ingestion (known as video-observed therapy (VOT)). The characteristics of the included studies are listed in table 2. Medication sleeves with phone calls and digital pillboxes were assessed under real-world conditions for their ability to detect adherence during TB treatment while ingestible sensors, VOT (AI interpretation) and the mobile application were assessed for their technical performance under controlled conditions. Detailed descriptions of the DATs and reference standards used for performance assessment for all articles are provided in online supplemental table S4. Briefly, included articles used either isoniazid (INH) urine metabolite tests, rifampicin (RIF) urine colour tests, pill counts, DOT or healthcare provider-based adherence reports to assess DAT performance (table 2). 12 articles reported on persons treated for TB disease while 1 considered the performance of a digital pillbox in persons treated for TB infection. Eight articles assessed DAT performance per person^9^,^1233^ while five used medication dose as the unit of assessment.^37^,^41^ Technical performance of DATs was assessed using total medication doses summed across participants.^38^,^41^ Two studies compared pill counts and urine drug tests with the digital pillbox as their reference standard; we back-calculated performance parameters for the pillbox compared with pill counts and urine tests using the primary data they reported (table 2). One study investigating digital pillboxes (Huan *et al*,^34^ 2012) was translated from Simplified Chinese to English using Google Translate (Alphabet, Mountain View USA).^42^

**Table 2:** Characteristics of included studies assessing the performance of DATs for measuring TB medication adherence

Study ID	Country	Participants				Intervention			Reference test		
		Participants*	Age (years)	N†	HIV%	DAT	Duration	Adherence classification	Test	Frequency	Adherence classification
Scott *et al*^33^ 2023	China‡ South Africa‡ Spain§ USA§	TB infection	Median: 36 IQR: 27–49	665	1%	Digital pillboxes	3 months	≥11/12 doses recorded	Pill count	1×/person	≤1 dose remaining
Bionghi *et al*^37^ 2018¶	South Africa‡	MDR TB disease	Median: 42 IQR: 33.5–54	21	100%	Digital pillboxes	3 weeks	1 dose recorded	Pill count	3×/person	1 dose missing**
Huan *et al*^34^ 2012	China‡	DS TB disease	Mean: 46 SD: 17	319	NR	Digital pillboxes	1–6 months	1 dose recorded in prior 24 hours	RIF urine colour test	1×/person	Red colour change
van den Boogaard *et al*^35^ 2011	Tanzania††	PTB and EPTB disease	Mean: 41 SD: 14	37	44%	Digital pillboxes‡‡	6 months	100% or ≥95% of doses recorded	Pill count	12×/person	0 or ≤5% doses remaining§§
									INH urine test	4×/person	Purple/blue colour change§§
									RIF urine colour test	4×/person	Orange urine colour
Ruslami *et al*^36^ 2008	Indonesia††	PTB disease	Median: 32 Range: 16–84	30	NR	Digital pillboxes‡‡	4 weeks	100% of doses recorded¶¶	Pill count	2×/person	0 doses remaining
Subbaraman *et al*^12^ 2021***	India††	DS PTB and EPTB disease	Median: 35 IQR: 25–45	608	47%	Medication sleeves with phone calls	1–6 months	Adherence: 2 or 3 reported dose over the 2 days prior to the urine test and the day of the test Non-adherence: 0 or 1 reported dose over the 2 days prior to urine test and the day of the test	INH urine test	1×/person	Purple/blue or green colour change§§
Thomas *et al*^9^ 2020	India††	DS PTB and EPTB disease	Median: 35 Range: 18–83	597	48%	Medication sleeves with phone calls	1–6 months	Adherence: 1 dose reported in the prior 6h to 48h Non-adherence: No dose reported in the prior 72 hours	INH urine test	1×/person	Purple/blue or green colour change§§
Efo *et al*^10^ 2021	Tanzania††	DS TB disease	Range: 25–44	197	NR	Medication sleeves with phone calls	6 months	Adherence: 1 dose reported in the prior 48 hours Non-adherence: No dose reported in the prior 48 hours	INH urine test	1×/person	Purple/blue or green colour change§§
Alacapa *et al*^11^ 2020†††	Philippines††	NR	NR	103	NR	Medication sleeves with phone calls	~3 months	Adherence: Dose reported in the prior 48h¶¶ Non-adherence: No dose reported in the prior 48 hours	INH urine test	1×/person	Purple/blue or green colour change§§
Browne *et al*^38^ 2019	USA§	DS TB disease	Mean: 43 SD: 17	77	NR	Ingestible sensors	2–3 weeks	1 detected dose	DOT	9×/person	1 dose ingested**
Belknap *et al*^39^ 2013	USA§	TB disease‡‡‡	Median: 44 Range: 22–79	30	10%	Ingestible sensors	2–3 weeks	1 detected dose	DOT	10×/person	1 dose ingested**
Sekandi *et al*^40^ 2023	Uganda§§§	DS TB disease	Mean: 31 Range: 19–50	51	28%	Other: VOT (AI result)	N/A¶¶¶	1 detected dose	VOT (Provider result)	10×/person	Research team identifies ingestion in VOT**
Goodwin *et al*^41^ 2022†††	Argentina‡	DS TB disease	NR	NR	NR	Other: Mobile App (Software result)	N/A¶¶¶	1 detected dose	Mobile App (Provider result)	NR	Treatment supporter/research staff detects colour change in photo of INH Urine Test.**

* If not specified, resistance/susceptibility details or TB type were not indicated.

† N indicates the number of participants analysed as part of DAT performance. Participant characteristics may be different among this specific group but were not reported in the text.

‡ Upper-middle-income country.

§ High-income country.

¶ Inpatient cohort.

** Study considered number of total medication doses across participants rather than adherence per participant’s treatment cycle.

†† Lower-middle-income country.

‡‡ Digital pillbox was used as the reference standard of the published study. Performance outcomes of the pillbox were calculated from originally published values on sensitivity, specificity, PPV and NPV.

§§ Colour change indicates the time of last medication ingestion: purple/blue (<24 hours ago), green (24–48 hours ago), yellow (>48 to 72 hours ago).

¶¶ Assumed based on study information.

*** Same cohort as Thomas *et al,*^9^ 2020.

††† Abstract from the Union World Conference on Lung Health

‡‡‡ Potentially INH-resistant TB in some clients.

§§§ Low-income country.

¶¶¶ Not applicable due to batch submitted videos or photos being used to assess performance of the AI or software.

AI, artificial intelligence; DATs, digital adherence technologies; DOT, directly observed therapy; DR, drug-resistant; DS, drug-sensitive; EPTB, extra-pulmonary tuberculosis; INH, isoniazid; MDR, multidrug-resistant; N, number of participants; N/A, not applicable; NPV, negative predictive value; NR, not reported; PPV, positive predictive value; PTB, pulmonary tuberculosis; RIF, rifampicin; TB, tuberculosis; VOT, video-observed therapy.

Assessment of adherence by the reference standard was conducted anywhere from once to twelve times over participants’ treatment; the duration of DAT use ranged from 2 weeks to 6 months (table 2). For two studies, authors were contacted with requests to complete missing data, but they did not reply. Six of the full articles reported some but not all applicable performance parameters (sensitivity, specificity, PPV, NPV, accuracy; online supplemental table S5). While two studies estimated area under the receiver operating characteristic (ROC) curve (online supplemental table S5), none provided ROC curves, likelihood ratios or estimated agreement. When possible, we calculated missing performance parameters from the data provided (online supplemental table S5). A meta-analysis pooling performance estimates was not conducted given the small number and marked heterogeneity of the studies retrieved. Only one^38^ of five studies^37^,^41^ that analysed repeated observations of individuals accounted for clustering in the primary analysis. We did not generate summary ROC curves to demonstrate joint sensitivity and specificity, given the small number of studies per DAT.

### Performance of DATs under real-world conditions

#### Medication sleeves with phone calls (‘99DOTS’)

**Figure 2:**
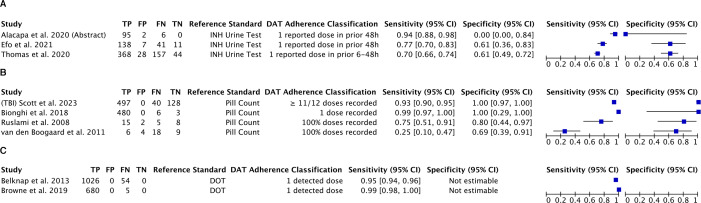
Forest plot of the sensitivity and specificity of (**A**) medication sleeves with phone calls (‘99DOTS’), (**B**) digital pillboxes and (**C**) ingestible sensors. (**A**) Isoniazid (INH) urine test was used as the reference standard. Digital adherence technology (DAT) adherence was recorded using ‘patient-reported doses’, wherein dose reporting relies only on calls made by the person receiving treatment. (**B**) Pill count was used as the reference standard. Results are depicted for persons with tuberculosis (TB) disease or infection. Bionghi *et al’*s cohort^37^ was an inpatient cohort. Scott *et al*^33^ used a cohort of persons with TB infection (TBI). In Ruslami *et al*^36^ and van den Boogaard *et al*,^35^ the digital pillbox was used as the reference standard. Number of true positives (TPs), false positives (FPs), false negatives (FNs) and true negatives (TNs) were back-calculated from the article’s primary data on pill count sensitivity and specificity, and number of events. (**C**) Directly observed therapy (DOT) was used as the reference standard. Sensitivity was calculated using the reported positive detection accuracy of each study (# of ingestible sensors detected/# of ingestible sensors ingested). Specificity was not estimable due to the lack of false positives and true negatives. Browne *et al*^38^ confidence intervals (CIs) account for within-individual clustering.

Three studies investigated the performance of medication sleeves with phone calls for measuring TB medication adherence, compared with unannounced INH urine tests either in the clinic or at a home visit (table 2). They generally classified adherence as any dose reported within 48 hours before the urine test and non-adherence as no reported dose within the previous 48 or 72 hours before the urine test.^9^,^11^ All studies investigating this DAT were conducted in lower-middle-income countries. The sensitivity of participants’ dose reports (patient-reported doses) for detecting adherence ranged from 70% to 94% while the specificity of participants’ dose reports for detecting non-adherence ranged from 0% to 61% (figure 2A).

Two articles on medication sleeves with phone calls, using data from the same cohort, showed that sensitivity and specificity vary depending on the approach used to classify DAT adherence. For example, when ‘non-adherence’ was defined as a person with TB not reporting any medication dose during the 72 hours prior to INH urine testing,^9^ the specificity was lower than when ‘non-adherence’ was defined as a person reporting less than either two or three medication doses in the 72 hours prior to the home visit (table 3).^12^

**Table 3:** Performance of medication sleeves with phone calls and digital pillboxes under real-world conditions against a reference standard

Study ID	DAT adherence classification	Reference Standard	N	TP	FP	FN	TN	Sensitivity (95% CI)	Specificity (95% CI)	PPV (95% CI)	NPV (95% CI)	Accuracy (95% CI)
**Medication sleeves with phone calls (‘99DOTS’)**												
Subbaraman *et al*^12^ 2021*	2 or 3 reported doses in 72 hours prior	INH urine test	608	330	20	211	47	61% (57 to 65)	70% (58 to 81)	94% (91 to 96)	18% (14 to 23)	62% (58 to 66)
			608†	482	45	59	22	89% (86 to 91)	33% (22 to 45)	91% (89 to 94)	27% (18 to 38)	83% (80 to 86)
Efo *et al*^10^ 2021	1 reported dose in prior 48 hours	INH urine test	197	138	7	41	11	77% (70 to 83)	61% (36 to 83)	95% (90 to 98)	21% (12 to 34)	76% (69 to 81)
Thomas *et al*^9^ 2020	1 reported dose in prior 6–48 hours	INH urine test	597	368	28	157	44	70% (66 to 74)	61% (48 to 72)	93% (91 to 95)	21% (18 to 25)	69% (65 to 72)
			597†	446	44	79	28	85% (81 to 88)	39% (28 to 52)	91% (90 to 93)	26% (20 to 33)	79% (76 to 82)
			287‡	155	17	83	32	65% (59 to 71)	66% (51 to 79)	90% (86 to 93)	28% (23 to 34)	65% (59 to 71)
			310§	213	11	75	11	74% (68 to 79)	48% (26 to 70)	95% (93 to 97)	12% (7 to 18)	72% (67 to 77)
Alacapa *et al*^11^ 2020¶	1 reported dose in prior 48 hours	INH urine test	103	95	2	6	0	94% (88 to 98)	0% (0 to 84)	98% (93 to 100)	0% (0 to 46)	92% (85 to 97)
**Digital pillboxes**												
*TB disease*												
Bionghi *et al*^37^ 2018**	1 dose recorded	Pill count	21†† (489)	480	0	6	3	99% (97 to 100)	100% (29 to 100)	100% (99 to 100)	33% (7 to 70)	99% (97 to 100)
Huan *et al*^34^ 2012¶	1 dose recorded in prior 24 hours	RIF urine colour test	319	208	5	1	105	100% (97 to 100)	95% (90 to 99)	98% (95 to 99)	99% (95 to 100)	98% (96 to 99)
van den Boogaard *et al*^35^ 2011‡‡	≥95% doses recorded	Pill count	37	19	11	5	2	79% (58 to 93)	15% (2 to 45)	63% (44 to 80)	29% (4 to 71)	57% (39 to 73)
		INH urine test	37	18	12	4	3	82% (60 to 95)	20% (4 to 48)	60% (41 to 77)	43% (10 to 82)	57% (39 to 73)
		RIF urine colour test	37	8	22	1	6	89% (52 to 100)	21% (8 to 41)	27% (12 to 46)	86% (42 to 100)	38% (22 to 55)
	100% doses recorded	Pill count	37	6	4	18	9	25% (10 to 47)	69% (39 to 91)	60% (26 to 88)	33% (17 to 54)	41% (25 to 58)
		INH urine test	37	4	6	18	9	18% (5 to 40)	60% (32 to 84)	40% (12 to 74)	33% (17 to 54)	35% (20 to 53)
		RIF urine colour test	37	2	8	7	20	22% (3 to 60)	71% (51 to 87)	20% (3 to 56)	74% (54 to 89)	59% (42 to 75)
Ruslami *et al*^36^ 2008‡‡	100% doses recorded§§	Pill count	30	15	2	5	8	75% (51 to 91)	80% (44 to 97)	88% (64 to 99)	62% (32 to 86)	77% (58 to 90)
*TB Infection*												
Scott *et al*^33^ 2023	≥11/12 doses recorded	Pill count	665 (Total)	497	0	40	128	93% (90 to 95)	100% (97 to 100)	100% (99 to 100)	76% (69 to 82)	94% (92 to 96)
			513 (USA)	395	0	17	101	96% (93 to 98)	100% (96 to 100)	100% (99 to 100)	86% (78 to 91)	97% (95 to 98)
			57 (SA)	25	0	21	11	54% (39 to 69)	100% (72 to 100)	100% (86 to 100)	34% (19 to 53)	63% (49 to 76)
			65 (Spain)	50	0	2	13	96% (87 to 100)	100% (75 to 100)	100% (93 to 100)	87% (60 to 98)	97% (89 to 100)
			30 (China)	27	0	0	3	100% (87 to 100)	100% (29 to 100)	100% (87 to 100)	100% (29 to 100)	100% (88 to 100)

If not reported, values and binomial CIs were calculated from other reported performance data (sensitivity, specificity, PPV, NPV or number of TP, FP, FN and TN).

* Same cohort as Thomas *et al*,^9^ 2020.

† Patient and provider reported doses (patient-reported doses relies only on calls made by the participant. For patient and provider reported doses, if no call is made after 24 hours, healthcare workers are supposed to contact the participant and report whether these doses were taken based on verbal report).

‡ Subpopulation of people with HIV within this cohort study.

§ Subpopulation of people without HIV within this cohort study.

¶ Abstract from the Union World Conference on Lung Health

** Inpatient cohort.

†† 489 total doses, shown in parentheses, were assessed for performance across 21 participants.

‡‡ The digital pillbox was used as the original reference standard. Number of TPs, FPs, FNs and TNs were back-calculated from the article’s primary data on pill count or urinalysis sensitivity and specificity, and number of events.

§§ Assumed based on study information.

DAT, digital adherence technology; FN, false negative; FP, false positive; INH, isoniazid; N, number of participants; NPV, negative predictive value; PPV, positive predictive value; RIF, rifampicin; SA, South Africa; TN, true negative; TP, true positive.

The same authors also examined dose verification added by providers, who telephoned persons who had not phoned in their doses on a given day. When provider-reported doses were added, the sensitivity of combined patient and provider dose reports for detecting adherence increased from 70% (95% CI 66% to 74%) to 85% (95% CI 81% to 88%), but specificity for detecting non-adherence decreased from 61% (95% CI 48% to 72%) to 39% (95% CI 28% to 52%) (table 3).^9^

Estimated overall accuracy ranged from 69% to 92% (table 3). Nonetheless, the NPV, or likelihood of a participant being non-adherent by the urine test among those classified as being non-adherent by the DAT, was consistently low (range 0%–26%). In other words, non-reporting of a dose by a person with TB often did not mean that they had in fact missed it—and instead often signalled limited ongoing engagement with the technology.

#### Digital pillboxes

The five studies reporting on digital pillboxes used varying methods and were conducted in countries with differing income levels (table 2). Two used the digital pillbox as their reference standard against multiple index tests, so we back-calculated performance estimates from their reported values (online supplemental table S5).^35 36^ One assessed performance in an inpatient cohort^37^ while one focused on TB infection.^33^ Four reports included pill counts conducted at clinic visits as at least one of their reference standards^3335^,^37^ while one used unannounced urine colour testing for rifampin at home visits^34^ (table 2).

When compared against pill count for persons with TB disease, three studies reported digital pillbox sensitivities for overall perfect adherence ranging from 25% to 99% while specificity for detecting non-adherence was higher, ranging from 69% to 100% (figure 2B).^35^,^37^ The report which only used urine colour testing for rifampin estimated the sensitivity and specificity of digital pillboxes for dose detection within 24 hours as 100% (95% CI 97% to 100%) and 95% (95% CI 90% to 99%), respectively (table 3).^34^ In one article, when compared against pill count among persons treated for TB infection, digital pillboxes were very sensitive for adherence (93% (95% CI 90% to 95%)) and specific for non-adherence (100% (95% CI 97% to 100%)) and generally remained so in subgroup analyses involving various study sites (table 3).^33^

For digital pillboxes, overall accuracy estimates ranged from 35% to 99% (table 3). As with the medication sleeves, NPVs were often poor, meaning that doses not reported by pillbox opening were not necessarily missed.

### Performance of DATs under controlled conditions

#### Ingestible sensors

Two reports from the USA addressed the performance of ingestible sensors for measuring TB medication adherence under controlled conditions (table 2).^38 39^ This involved determining if an on-body wearable sensor could detect the ingestible sensor placed on the medication, following its ingestion which was directly observed. The percentage of doses that were correctly classified as taken by the sensor technology (sensitivity) was at least 95% across studies (figure 2C, online supplemental table S6). Only sensitivity could be estimated.

#### Other DATs

Two reports assessed other DATs for their ability to measure TB medication adherence under controlled conditions. In one, a deep convolutional neural network was used to determine whether participants had ingested medication during videos submitted as part of a VOT intervention.^40^ The performance of the AI algorithm was compared with the research team’s interpretation of the same submitted videos and assessed using fivefold cross-validation, for five types of convolutional neural networks to extract features of the videos. The best-performing convolutional neural network had a mean sensitivity of 95% (SD 2.6) but a specificity of 55% (SD 6.5) (online supplemental table S6).

In the other report, a mobile application involved the participant’s uploading a photo of their INH urine test, and associated software then analysed the images to determine whether or not they indicated the presence of INH metabolites.^41^ The output from this reader software was compared with treatment supporter and research staff interpretation of the same urine test images. The sensitivity was 81% and 86% when compared with the treatment supporter and research staff interpretations, respectively, while the percentage of doses correctly classified by the technology as not taken (specificity) was 95% and 91% (online supplemental table S6).

### Subgroups and special populations

In an inpatient cohort of 21 individuals with both HIV and drug-resistant TB, digital pillboxes had high sensitivity and specificity (>99%) (table 3).^37^ In another study investigating medication sleeves with phone calls, a subgroup analysis of people with HIV treated for TB disease found that specificity was higher (66% (95% CI 51% to 79%)) among people with HIV (ie, more non-adherence was correctly detected) than among people without HIV (48% (95% CI 26% to 70%)) (table 3).^9^ Several other studies included persons living with HIV among their participants but did not report results separately for this group (table 2).^35 39^ Three articles included persons with both pulmonary and extrapulmonary TB, however, results were not reported separately (table 2).^9 12 35^ No report addressed persons younger than 18.

### Quality assessment

The risk of bias was high or unclear in at least two categories for all but one of the reports assessed (online supplemental figure S1A). Two reports could not be assessed for quality as the limited description of their methods precluded formal assessment of bias.^11 41^ Participant selection was a frequent source of potential bias since participants were rarely randomly sampled; in some cases, they had also explicitly consented to take part in DAT implementation projects,^10^ which could also introduce bias. Flow and timing were also frequent sources of potential bias because of participants missing from the analysis, and unclear or suboptimal timing of DAT performance assessment relative to reference standard measures. There were fewer concerns about applicability with respect to participant profiles, DATs used or reference standards (online supplemental figure S1B). Details of the quality assessment criteria are provided in online supplemental table S7.

### Publication bias

There was visual asymmetry in the funnel plot displaying DORs and ESSs, indicating possible publication bias (online supplemental figure S2). There were too few studies to permit formal quantitative evaluation of publication bias. Additionally, the ESSs do not account for within-individual clustering and are, therefore, overestimated in some cases.^37 40^ Several studies could not be included in the assessment of publication bias because of insufficient data to estimate the DORs.

### GRADE results

The findings suggested moderate certainty of evidence for the sensitivity of ingestible sensors because of consistent and precise results, a low risk of bias, but an inability to assess publication bias. The certainty of evidence was very low for medication sleeves with phone calls because of substantial risks of bias, and substantial variation in sensitivity and specificity across articles. Similarly, the certainty of evidence was very low for digital pillboxes. More details on the quality of evidence for each DAT can be found in online supplemental tables S8–S10 and online supplemental figure S3. A GRADE assessment was not conducted for the DATs using AI and reader software since these involved only one report each.

## Discussion

Available evidence addressing the performance of DATs for measuring TB medication adherence is limited in scope and quality. Among 13 reports which considered 5 types of DATs, there was substantial variation in reference standards, definitions of adherence and adherence classifications, which made it difficult to compare and summarise results. In general, studies of real-world DAT use suggested suboptimal performance of these technologies for measuring medication adherence. These findings are concordant with a previous review that assessed the accuracy of electronic monitoring devices for antiretroviral medication adherence.^43^ Some degree of measurement error is expected, to the extent that DAT adherence measures serve as proxies for medication ingestion. However, our results suggest substantial limitations to DAT performance under real-world conditions. Ingestible sensors and AI-based video-supported therapy, although not studied under real-world conditions, more closely approximate direct observation of ingestion, but the attendant expense and required infrastructure may limit their applicability in lower-income settings where most people with TB are treated. While a few studies suggested stronger performance of digital pillboxes, this was potentially biased by inpatient treatment^37^ or shorter course drug regimens.^33^

To understand the suboptimal performance of DATs during real-world implementation, it is important to consider that this reflects the ways in which people undergoing TB treatment do or do not engage with the technology. Engagement may be influenced by technical attributes of the DAT and the motivations, beliefs, social context and structural barriers faced by people with TB. Suboptimal engagement—which may result in under-reporting of true medication adherence (ie, reduced sensitivity, reduced NPV)—can be influenced by limited access to mobile networks and technology, shared cellphone use among multiple household members and inadequate education about the purpose and appropriate use of the DAT.^14^,^17^ With substantial under-reporting of medication adherence, healthcare providers may have difficulty identifying people who are truly experiencing non-adherence, may be unable to routinely reach out to all people with reported non-adherence (eg, via phone calls or home visits)^44^ and may begin to ignore digital adherence data because of performance concerns.^16 44^

Conversely, over-reporting of adherence (ie, reduced specificity, reduced PPVs)—in which people with TB or healthcare providers may report adherence via phone call or pillbox opening despite medication doses not actually ingested—may result from the desire by people with TB to conceal non-adherence or by the desire of healthcare providers to report optimal TB outcomes.^13^ Over-reporting may be more concerning, as healthcare providers and health systems may miss early identification of people who need intensified support towards optimal treatment outcomes. Indeed, one potential use of digital adherence monitoring is triage, to distinguish people who face important barriers to successful treatment, so that they can be offered more personalised support (psychological, social and financial).^45^ Poor specificity/dosing overestimation compromises the use of DATs for such triage.

Related to user engagement, acceptance and experience with DATs were assessed in five articles^1035 37^,^39^: most people with TB reported positive experiences and high acceptability. However, programmatic reports suggest more variable user experiences.^14^,^1744^ Indeed, two forthcoming reviews address barriers and facilitators for DAT implementation in greater detail.^46^

Several challenges limited our interpretation of DAT performance. The certainty around performance under programmatic conditions was very limited. Additionally, unclear participant flow and variable timing of tests, use of different pill-taking thresholds to define adherence, and of different windows for dose recording versus urine testing, add substantial complexity and make it even more difficult to summarise the evidence. One study evaluated digital pillboxes in an inpatient setting, which differs substantially from the settings where DATs would most likely be used and limits the relevance of the resulting data. Another used a method for documenting the presence or absence of rifampin in urine, which was not used in other studies, highlighting the importance of consistent reference standards. More generally, the use of urine tests as reference standards can be problematic, given known gaps in their performance. This includes suboptimal sensitivity for doses ingested over 24 hours before testing,^47^ and suboptimal specificity for doses ingested over 72 hours before testing.^12 48^ Similarly, while pill count is a standard method for determining adherence, pills missing during routine pill counts may not have been ingested. Improvements in flow and timing of the DATs and reference standards, as well as standardised measures of adherence would strongly improve the evidence base.

### Strengths and limitations

To our knowledge, this is the first systematic review focused specifically on the performance of DATs for measuring TB medication adherence. We used a wide-ranging, prespecified search strategy across multiple databases, without language restriction and with the potential inclusion of suitable reports from grey literature, including preprints. Our search was developed and conducted by an experienced health sciences librarian. Selection of reports and data extraction were performed rigorously, by two independent reviewers at every step. Whenever possible, we used primary data to estimate any relevant performance parameters that authors had not reported. We also assessed the quality and robustness of the available evidence.

For logistical reasons, we could not systematically search for and retrieve abstracts other than those presented at the Union World Conferences on Lung Health. Some reports did not include sufficient data to permit estimation of all performance parameters of interest. Additionally, there may be an underestimation of variance in instances where clustering was not considered (ie, for repeated measurements per individual). We did not identify any reports evaluating the performance of doses observed by video against urine tests or pill counts, although video-supported treatment has been used increasingly in high-income countries.^49^,^51^ Most reports focused on outpatients with drug-susceptible TB disease, except one that involved persons with TB infection,^33^ and one that involved an inpatient cohort of persons with HIV and multidrug-resistant TB disease.^37^ Our ability to draw specific conclusions for the latter two groups is therefore limited. Finally, given the heterogeneity of study methods, technologies and results, we did not formally pool or meta-analyse their data.

### Changes to the protocol

Changes were made to our preregistered protocol in PROSPERO as follows. We additionally searched Europe PMC for preprint articles relevant to our review. We did not anticipate the use of DOT, or provider-based DAT decisions as reference standards for quantifying DAT performance, and therefore, added them as possible comparators. We also did not anticipate articles that used the DAT as the reference standard. For those reports, we back-calculated the published results to provide us with the performance of the DAT as the index test.

## Conclusions

The performance of digital technologies for measuring TB medication adherence appears variable and limited. The use of digital pillboxes may be preferred but this was not consistent across articles. Substantial over-reporting and under-reporting of medication doses was documented for the DATs studied under real-world conditions (ie, digital pillboxes, medication sleeves with phone calls). Accurate dose reporting is fundamental to the use of digital technologies to aid treatment adherence. Their use as alternatives to traditional direct observation is predicated on the concept that providers and health programmes can provide additional support to people who appear to be facing challenges in this regard. Hence, suboptimal performance of dose reports from DATs can potentially compromise their effectiveness, as well as programme efficiency. If the DAT fails to capture significant non-adherence, this leads to missed opportunities for intervention, and potentially even poorer treatment outcomes. On the other hand, if support and supervision are intensified for people wrongly labelled as poorly adherent by the DAT, this is a waste of limited programme resources.

The future evidence base will be strengthened by more consistent definitions and cutoffs for adherence, clearer and appropriate intervals between tests, and by more consistent use of one or more reference standards—for example, validated methods for pill counts, standardised timing and technique for urine tests. Future research should also address the interplay of specific technology, setting, user characteristics and user engagement with DAT performance. Additional studies examining the performance of asynchronous video observation and ingestible sensors in real-world conditions will also be important, as will further investigation of digital technologies to support newer treatment regimens for TB disease (including drug-resistant and extrapulmonary TB) and TB infection.

## Supplementary material

10.1136/bmjgh-2024-015633 online supplemental file 1

## Data Availability

Data are available in a public, open access repository.
